# Quality and safety investigation of commonly used topical cosmetic preparations

**DOI:** 10.1038/s41598-022-21771-7

**Published:** 2022-10-31

**Authors:** May Almukainzi, Lubna Alotaibi, Anfal Abdulwahab, Nada Albukhary, Areej M. El Mahdy

**Affiliations:** 1 Department of Pharmaceutical Sciences, College of Pharmacy, Princess Nourah bint Abdulrahman University, P.O. Box 84428, Riyadh, 11671 Saudi Arabia; 2 College of Pharmacy, Princess Nourah bint Abdulrahman University, Riyadh, Saudi Arabia; 3 Department of Microbiology and Immunology, Faculty of Pharmacy, Mansoura University, Mansoura, Egypt

**Keywords:** Drug regulation, Drug safety, Pharmaceutics, Natural hazards, Health care, Drug regulation

## Abstract

Cosmetic and personal care products are considered an essential part of our daily care routine; hence, these products must be stable and safe for human use. This study aimed to assess the quality and safety of the most common cosmetic preparations. To select the products to be tested, a cross-sectional survey was distributed featuring the most used types and brands of products. Based on 447 responses from both males and females with different ages and education levels, 21 products from different brands were selected and tested in terms of microbial load, heavy metal content, and organoleptic properties. Microbial contamination was investigated using the aerobic plate count method. Lead (Pb), aluminum (Al), cadmium (Cd), cobalt (Co), chromium (Cr), copper (Cu), manganese (Mn), nickel (Ni), zinc (Zn), iron (Fe), and arsenic (As) impurities were analyzed using an inductively coupled plasma mass spectrometer. The products included sunblock, lip balm, hand cream, hair cream, shampoo, cleanser, baby oil, baby powder, bar soap, hair dye, makeup, deodorant, hair serum, shaving gel, and toothpaste. Microbial contamination was found in 14 of the products, ranging between 1467.5 and 299.5 cfu/ml. The most commonly isolated microorganisms were *Staphylococcus aureus* and *Bacillus* species. Most of the tested products showed metal impurities, with toothpaste having the highest concentrations of Pb, Cr, As, Cu and Ni. The samples did not show lumps or discoloration, did not have characteristic odors, and had pH values ranging from 6.90 to 8.10. The continuous usage of such products could lead to serious negative consequences. As a result, ensuring the quality of cosmetic products is critical. Regulatory authorities are required to enforce strict legislation on cosmetic manufacturing to assess and ensure the quality and safety of the products before they reach consumers.

## Introduction

Cosmetic is a Greek word that means ‘adorn’; these products are derived from natural and synthetic resources to improve the appearance and/or function of the skin. Historically, numerous compounds from natural sources have been used as cosmetics, such as henna, lavender, peppermint, rosemary, cedar, rose, aloe, olive oil, sesame oil, and almond oil^1^. Currently, cosmetic products are considered an essential part of the daily care routine^2^. Cosmetic products can be used dermally, orally or be inhaled. Topical applications are the most common route of cosmetics exposure^3^. Products that fall under this category can be in the form of creams, gels, ointments, pastes, suspensions, lotions, foams, sprays, and aerosols^4^. Topical cosmetic products, commonly known as personal care products, are among the most consumed products globally; their accessibility and widespread availability make their purchase easier than ever. These products are intended to be rubbed, sprinkled, poured, or sprayed for cleansing, beautifying, adding attractiveness or modifying the physical appearance to several body parts, such as the hair, skin, and nails^5^. Therefore, these preparations must be effective, stable, and safe for human use.

Quality assurance and monitoring are significant practices that guarantee that cosmetics preparations are safe and effective^6,7^. A desired quality in the finished product is granted when the quality is controlled and maintained in each step from acquiring the raw material until the product reaches the consumer^6,8^. As cosmeceutical and cosmetic products are continuously produced and introduced into the market, quality concerns have increased^3^.

One serious concern with the quality and safety of topical cosmetic products in different parts of the world is microbial contamination^9^. The high percentage of the water contents in these preparations make it an ideal condition for bacteria growth. For example, a study that investigated the quality of topical cosmetic products of eleven herbal ointments commonly found on the Ghanaian market showed some quality deviations in these products^10^; the study found that the majority of the examined products were contaminated by different kinds of fungi and bacteria^10^. Another study investigated 93 commercially available cosmetic products on the Turkish market, including shampoos, shaving creams, moisturizing products, face care products, and makeup removers^11^. The microbiological analysis tests on these products showed high gram-negative and positive bacterial contamination^11^.

An additional serious concern with cosmetics is heavy metal contamination, such as aluminum (Al), iron (Fe), zinc (Zn), chromium (Cr), manganese (Mn), copper (Cu), nickel (Ni), arsenic (As), lead (Pb), cadmium (Cd), cobalt (Co) and many others. Topical cosmetic preparations can act directly on the skin and indirectly by penetrating the skin through systemic circulations. Therefore, harmful consequences of heavy metals contaminations can affect different body organs. This is especially true when these cosmetics are used frequently or when applied on a large surface area of the body as sunscreen and motorizing creams. Although heavy metals such as Pb, As, and Cd have been banned as intentional ingredients in cosmetic products, metal impurities can still be found^12^. A study that assessed some toxic heavy metals (Pb, Cd, Cr, and mercury) in six different cosmetics creams collected from a local Bangladesh market found that the Pb and Cd contents were high in all the tested samples^3^. A more recent study aimed to determine the concentrations of Co and Pb in four different types of cosmetic products using 41 brands available in Saudi Arabia's local markets and found that the contents of Co were very high^13^.

This study aims to assess the most commonly used topical cosmetic products and examine if these preparations are up to standard by assessing their organoleptic properties, microbial load and heavy metal impurities.

## Materials and methods

### Sample selection

The Institutional Review Board confirmed that the study proposal complied with national regulations, and the research was given "exempt" status (Registration Number# 21-0330 of Princess Noura University, Riyadh, Saudi Arabia).

To select the most common products, a closed- and open-ended questionnaire survey was distributed online to the public via social media applications. Consent was obtained from all the participants as the questionnaire started with a consent statement for participants to continue the survey. After the demographic questions, the first part of the survey asked about the frequent use of cosmetics and personal care products such as lotions, creams, toothpaste, shampoo, deodorant, sunblock, body powder, soap, face cleanser, shaving gel, whitening creams, makeup products and hair dyes. Then, the next part of the questions aimed to specify the most common brand for each product.

Based on the survey results, 21 samples of commonly used topical cosmetic products were purchased from local markets and drug stores. The product information is listed in Table 1.

**Table 1:** Topical cosmetic products used in the study.

Product code	Product type	Product constituents (according to the package)
1	Sunblock cream	Aqua, homosalate, butyl methoxydibenzoylmethane, ethylhexyl salicylate, ethylhexyl triazone, butylene glycol dicaprylate/dicaprate, alcohol denat., bis-ethylhexyloxyphenol methoxyphenyl triazine, phenylbenzimidazole sulfonic acid, silica, cyclomethicone, tapioca starch, behenyl alcohol, cetearyl alcohol, methylpropanediol, glycerin, glycyrrhiza inflata root extract, tocopherol acetate, sodium stearoyl glutamate, xanthan gum, carbomer, acrylates/c10-30 alkyl acrylate crosspolymer, dimethicone, sodium chloride, sodium hydroxide, trisodium EDTA, ethylhexylglycerin, phenoxyethanol, linalool, limonene, benzyl alcohol, alpha-isomethyl ionone, geraniol, citronellol, coumarin, perfume
2	BB cream	Octinoxate 3%, titanium dioxide 4.7%. inactive ingredients: water, isononyl isononanoate, isohexadecane, glycerin, alcohol denat., peg-20 methyl glucose sesquistearate, methyl glucose sesquistearate, acetyl palmitate, nylon-12, cyclohexasiloxane, propylene glycol, hydrogenated polyisobutene, stearyl alcohol, magnesium aluminum silicate, phenoxyethanol, caprylyl glycol, lithium magnesium sodium silicate, disodium EDTA, linalool, benzyl salicylate, limonene, caffeine, ascorbyl glucoside, benzyl alcohol, geraniol, cellulose acetate butyrate, polyphosphorylcholine glycol acrylate, citral, ammonium polyacryloyldimethyl taurate, polyvinyl alcohol, sodium chloride, butylene glycol, sodium hyaluronate, fragrance; may contain: titanium dioxide, iron oxides
3	Lip balm	octyldodecanol, *Ricinus communis* seed oil, cera alba, cocoglycerides, bis-diglyceryl polyacyladipate 2, helianthus annuus seed cera, cetearyl alcohol, hydrogenated castor oil, acetyl palmitate, *Simmondsia chinensis* seed oil, ascorbyl. palmitate, tocopherol, aqua, glycerin, *Rubus idaeus* juice, sodium benzoate, potassium sorbate, BHT, aroma, ci 77891, ci 15850
4	Hand cream	Aqua, glycerin, potassium lactate, dimethicone, tapioca starch, cetearyl alcohol, lactic acid, ethylhexyl methoxycinnamate, glyceryl hydroxystearate, olea europaea (olive) fruit oil, ammonium acryloyldimethyltaurate/vp copolymer, arachidic acid, BHT, butyl
5	Hair cream	water, sodium laurel sulfate, sodium laureth sulfate, sodium chloride, diacetyl glycol, citric acid, sodium citrate, sodium sulfate (sulphate), fragrance, dimethicone, sodium benzoate, diamine ethylene tetra-acetylene acid, poly quaternium 6, panthenol, panthenyl ethyl ether, methylchloroisothyzozolenone, methylethyzoethyazole
6	Shampoo	water, sodium laureth sulfate, dimethiconol, cocamidopropyl betaine, sodium chloride, glycerin, perfume, carbomer, sodium hydroxide, tetrasodium EDTA, tea-dodecylbenzenesulfonate, guar hydroxypropyltrimonium chloride, tea-sulfate, citric acid, amodimethicone, hydroxypropyl methylcellulose, mica, DMDM hydantoin, peg-45m, phenoxyethanol, sodium benzoate, paraffinum liquidum, lysine HCl, trideceth-12, cetrimonium chloride, *Orbignya speciosa* kernel oil, *Astrocaryum murumuru* seed butter, ppg-9, *Argania spinosa* kernel oil, panthenol, magnesium nitrate, dimethyl palmitamine, methylchloroisothiazolinone, silica, BHT, magnesium chloride, methylisothiazolinone, iodopropynyl butylcarbamate, pantolactone, acetic acid, TBHQ, butylphenyl methylpropional, linalool, citronellol, hexyl cinnamal, benzyl salicylate, alpha-isomethyl ionone, ci 77891, ci 17200, ci 42090, ci 19140
7	Facial cleanser	Water, sodium c14 16 olefin sulfonate, cocamidopropyl betaine, sodium c12 15 pareth 15 sulfonate, salicylic acid, linoleamidopropyl pg dimonium chloride phosphate, disodium EDTA, propylene glycol, FD&C yellow 5, FD&C red 40, fragrance
8	Baby oil	Paraffin oil light, perfume
9	Baby powder	Talcum powder, zinc oxide, salicylic acid, menthol & perfume
10	Bar soap	Sodium palmate, aqua, sodium palm kernelate, glycerin, parfum, sodium chloride, titanium dioxide, lauric acid, citric acid, tetrasodium, etidronic acid, sericin, *Jasminum officinale* flower extract, *Rose gallica* flower extract, *Nelumbium speciosum* flower oil, *Prunus amygdalus* dulcis oil, *Cymbopogon martini* oil, *Mentha arvensis* leaf oil, peg-40 hydrogenated castor oil, phenoxyethanol, disodium distyrylbiphenyl disulfonate, alpha-isomethyl ionone, butylphenyl propional, citronellol, coumarin, geraniol, hexyl cinnamal, limonene, linalool, ci74169
11	Hair dye	hexadimethrine chloride, glycol distearate, polyquaternium-2, ethanolamine, silica dimethyl silylate, p-phenylenediamine, ci 77891/titanium dioxide, 2,4-diaminophenoxyethanol HCl, m-aminophenol, ascorbic acid, sodium metabisulfite, dimethicone, pentasodium pentetate, N,N-bis(2-hydroxyethyl)-p-phenylenediamine sulfate, parfum/fragrance, carbomer, resorcinol, *Vitis vinifera* seed oil/grape seed oil. f.i.l. d35182/4. Fruit oil concentrate: paraffinum liquidum/mineral oil, *Vitis vinifera* seed oil/grape seed oil, parfum/fragrance, tocopherol, *Rosmarinus officinalis* (rosemary) extract. f.i.l. d25829/5. Rinse-out conditioner: aqua/water, cetearyl alcohol, *Elaeis guineensis* oil/palm oil, behentrimonium chloride, *Pyrus malus* extract/apple fruit extract, glycerin parfum/fragrance, stearamidopropyl dimethylamine, niacinamide, pyridoxine HCl, *Butyrospermum parkii* butter/shea butter, citric acid, olea europaea oil/olive fruit oil, saccharum officinarum extract/sugar cane extract, benzyl alcohol, chlorhexidine dihydrochloride, *Persea gratissima* oil/avocado oil, *Ribes nigrum* oil/black currant seed oil, linalool, citrus medica Limonum peel extract/lemon peel extract, ci 19140/yellow 5, *Camellia sinensis* extract/*Camellia sinensis* leaf extract, ci 15985/yellow 6. f.i.l. d35637/3. Nutrisse developer: aqua/water, hydrogen peroxide, cetearyl alcohol, trideceth-2 carboxamide mea, ceteareth-25, glycerin, pentasodium penetate, sodium stannate, tetrasodium pyrophosphate, phosphoric acid. f.i.l. d12836/26
12	Makeup powder	talc, mica, aluminum starch octenylsuccinate, magnesium stearate, isocetyl stearoyl stearate, octyldodecyl stearate, ethylhexylglycerin, aluminum hydroxide, phenoxyethanol, ci 15850 (red 6 lake, red 7 lake), ci 77491, ci 77499 (iron oxides)
13	Makeup mousse cream	cyclopentasiloxane, dimethicone, dimethicone crosspolymer, phenyl trimethicone, silica, talc, tribehenin, cyclohexasiloxane, c30-45 alkyl methicone, c30-45 olefin, triethoxycaprylylsilane, methicone, paraffinum liquidum (mineral oil), phenoxyethanol, methylparaben, ethylparaben, ci 77491 (iron oxides), ci 77492 (iron oxides), ci 77499 (iron oxides), ci 77891 (titanium dioxide)
14	Mascara	g3311/2: talc, triisostearin, hydrogenated polydecene, magnesium stearate, synthetic wax, calcium sodium borosilicate, hdi/trimethylol hexyllactone crosspolymer, synthetic fluorphlogopite, caprylyl glycol, silica, calcium aluminum borosilicate, perlite, tin oxide, alumina, magnesium silicate, [+ /− may contain/peut contenir, mica, ci 77891/titanium dioxide, ci 77491, ci 77492, ci 77499/iron oxides, ci 77400/bronze powder, ci 77742/manganese violet, ci 77000/aluminum powder, ci 77007/ultramarines, ci 77163/bismuth oxychloride, ci 75470/carmine, ci 77510/ferric ferrocyanide, ci 19140/yellow 5 lake, ci 77510/ferric ammonium ferrocyanide, ci 77400/copper powder, ci 77288/chromium oxide greens, ci 16035/red 40 lake, ci 42090/blue 1 lake], d130837/5
15	Deodorant	Aqua, aluminium chlorohydrate, glyceryl stearate, cetearyl alcohol, peg-20 stearate, glycerin, parfum, paraffinum liquidum, titanium dioxide, alpha-isomethyl ionone, benzyl alcohol, benzyl benzoate, benzyl salicylate, butylphenyl methylpropional, citronellol, geraniol, hexyl cinnamal, hydroxycitronellal, isoeugenol, limonene, linalool, tocopherol
16	Oil	Cyclopentasiloxane, dimethiconol, fragrance ingredient, ethylhexyl methoxycinnamate, BHT
17	Shaving cream	Water, glycerin, palmitic acid, triethanolamine, isopentane, glyceryl oleate, stearic acid, fragrance, isobutane, sorbitol, hydroxyethylcellulose, peg-90m, pef-23m, *Theobroma cacao* (cocoa) seed butter, blue 1
18	Lipstick	Polybutene, octyldodecanol, hydrogenated polyisobutene, caprylic/capric triglyceride, helianthus annuus (sunflower) seed wax, ozokerite, c12-15 alkyl benzoate, bis-diglyceryl polyacyladipate-2, polyethylene, hydrogenated polycyclopentadiene, tocopherol, pentaerythrityl tetra-di-t-butyl hydroxyhydrocinnamate, benzyl alcohol, aroma (flavor), linalool, ci 15850 (red 7 lake), ci 42090 (blue 1 lake), ci 77491 (iron oxides), ci 77492 (iron oxides), ci 77891 (titanium dioxide)
19	Toothpaste	Sodium fluoride 0.32%(1450 ppm of f), sorbitol, aqua, hydrate silica, sodium lauryl sulfate, aroma, ped-12, cellulose gum, sodium saccharin, limonene, ci 74260, ci42090
20	Toothpaste	Calcium carbonate, aqua (water), sorbitol, hydrated silica, sodium lauryl sulfate, sodium monofluorophosphate, armoma (flavour), cellulose gum, potassium citrate, trisodium phosphate, sodium saccharin, phenylcarbinol, glycerin, limonene, ci 12490./
21	Lotion	Aqua, paraffinum liquidum, isohexadecane, glycerin, isopropyl palmitate, peg-40 sorbitan perisostearate, cera microcristallina, polyglyceryl-3 diisostearate, prunus amygdalus dulcis oil, tocopherol, magnesium sulfate, sodium citrate, citric acid, tocopherol acetate, potassium sorbate, linalool, limonene, benzyl alcohol, geraniol, citronello, alpha isomethyl ionone, benzyl benzoate, perfume

### Microbial load testing

Microbial analysis was performed according to analytical bacteriological methods^14^.

#### Media

- Nutrient broth (13 g of nutrient broth powder in 1000 ml of distilled water).
- Nutrient agar (28 g of nutrient agar powder in 1000 ml of distilled water).
- MacConkey agar (55.07 g of MacConkey agar power in 1000 ml of distilled water).
- Potato dextrose agar (39 g of potato dextrose powder in 1000 ml of distilled water).
- etrimide agar (suspend 46.7 g of cetrimide agar powder in 1000 ml of distilled water and add 10 ml of glycerol).
- Simmons’ citrate agar (24 g of Simmons’ citrate agar in 1000 ml of distilled water).
- Mannitol salt agar (30 g of mannitol salt agar in 1000 ml of distilled water).

All media were sterilized in an autoclave immediately after preparation and poured directly into sterilized petri dishes.

#### Sample preparation

Samples were analyzed as soon as they were opened from the original containers and were kept at room temperature. Sample preparation was carried out in a sterilized area with 70% alcohol, near a flame and using sterilized equipment (pipettes, tips, test tubes). The samples were prepared by taking 1 g of each product, diluting it in 2 ml of nutrient broth and then incubating it in a Heratherm IGS60 CO_2_ incubator for 24 h. Samples, such as creams and oil-based products, needed to be more dilute, so 1 ml of Tween 20 was added to the samples. Using a sterilized pipette tip, 20 μl of solution was withdrawn from each of the samples, plated in duplicate on nutrient agar dishes and then incubated for 24 h.

For samples that showed growth, a sterilized tip was swiped on the colonies, dipped in a tube with 2 ml of nutrient broth and incubated for 24 h. 20 μl were withdrawn from each test tube, and the streak plate method was performed on all the Petri dishes using MacConkey, potato dextrose, cetrimide, Simmons’ citrate, and mannitol salt and then incubated for 72 h. After incubation, sterilized tips were swiped on the sample that showed growth on potato dextrose and examined under a microscope to test for the presence of fungi.

#### Enumeration step

A sterilized tip was swiped on the grown colonies of the first nutrient agar that showed growth and transferred to 2 ml of new nutrient broth. The tubes were vortexed and then incubated for 24 h. A serial dilution method was performed for all the tubes by withdrawing 500 μL from all 10^1^ tubes and adding it promptly to each newly prepared nutrient agar dish; the tubes were then vortexed to mix the sample with the agar. The contents were left to solidify at room temperature, and then the dishes were incubated for 24 h. Colonies were counted according to the plate count method.

### Metal sample preparation and digestion

Sample preparation was performed following elemental impurity procedures in the <232>, USP 40, NF 35^15^ method. The samples were first digested in a concentrated acid using a closed vessel digestion apparatus. The ratio of the acid mixture used depended on the sample matrix (Table 2).

**Table 2:** Ratios of the acid mixture used for sample preparation for metal impurity detection.

	Solvent volume (ml)			Sample Weight (mg)
	HNO_3_ (69%)	H_2_O_2_ (30%)	HF (37%)	
1	10	×	×	250
2	10	×	×	250
3	10	×	×	250
4	10	×	×	250
5	10	×	×	250
6	10	×	×	300
7	10	×	×	300
8	5	3	×	500
9	7	2	×	400
10	10	×	×	300
11	10	×	×	250
12	6	×	0.50	500
13	6	×	0.50	500
14	6	×	0.50	500
15	10	×	×	500
16	10	2	×	500
17	10	2	×	500
18	6	×	0.50	500
19	2	×	2	200
20	2	×	2	200
21	10	×	×	250

Elemental impurities were detected using inductively coupled plasma–mass spectrometry (ICP–MS). The ICP‒MS calibration curves of the eleven heavy metals were constructed using the blank and standards 5, 15, 30, 70, and 150 ppb for each sample from the standard solution. The elements in the calibration curves showed linearity, and the coefficient of determination values for all elements ranged from 0.9995 to 1, thus showing good fit into the respective curve. The concentrations of the impurities Al, Fe, Zn, Cr, Mn, Cu, Ni, As, Pb, Cd, and Co were determined.

### Organoleptic testing

Samples were visually inspected for any lumps, color and odors. The products were rubbed on the skin to test the texture; additionally, pH was determined by using a digital pH meter.

### Guideline

No experiments on humans and/or the use of human tissue sample, and the manuscript file includes a statement identifying the institutional committee approving the experiments. All methods were carried out in accordance with relevant guidelines and regulation.

## Results

**Figure 1:**
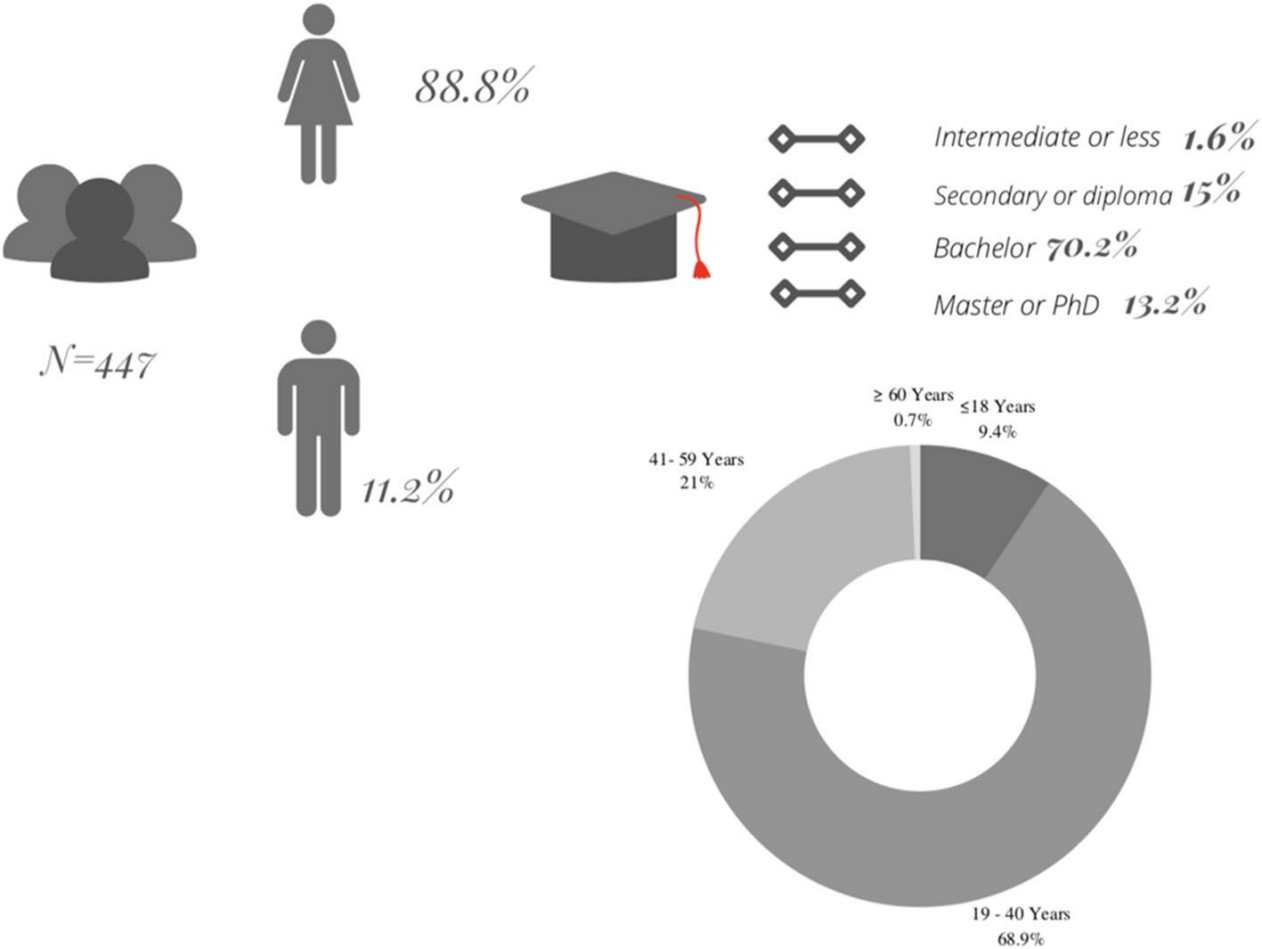
Demographics of the survey participants**.**

**Figure 2:**
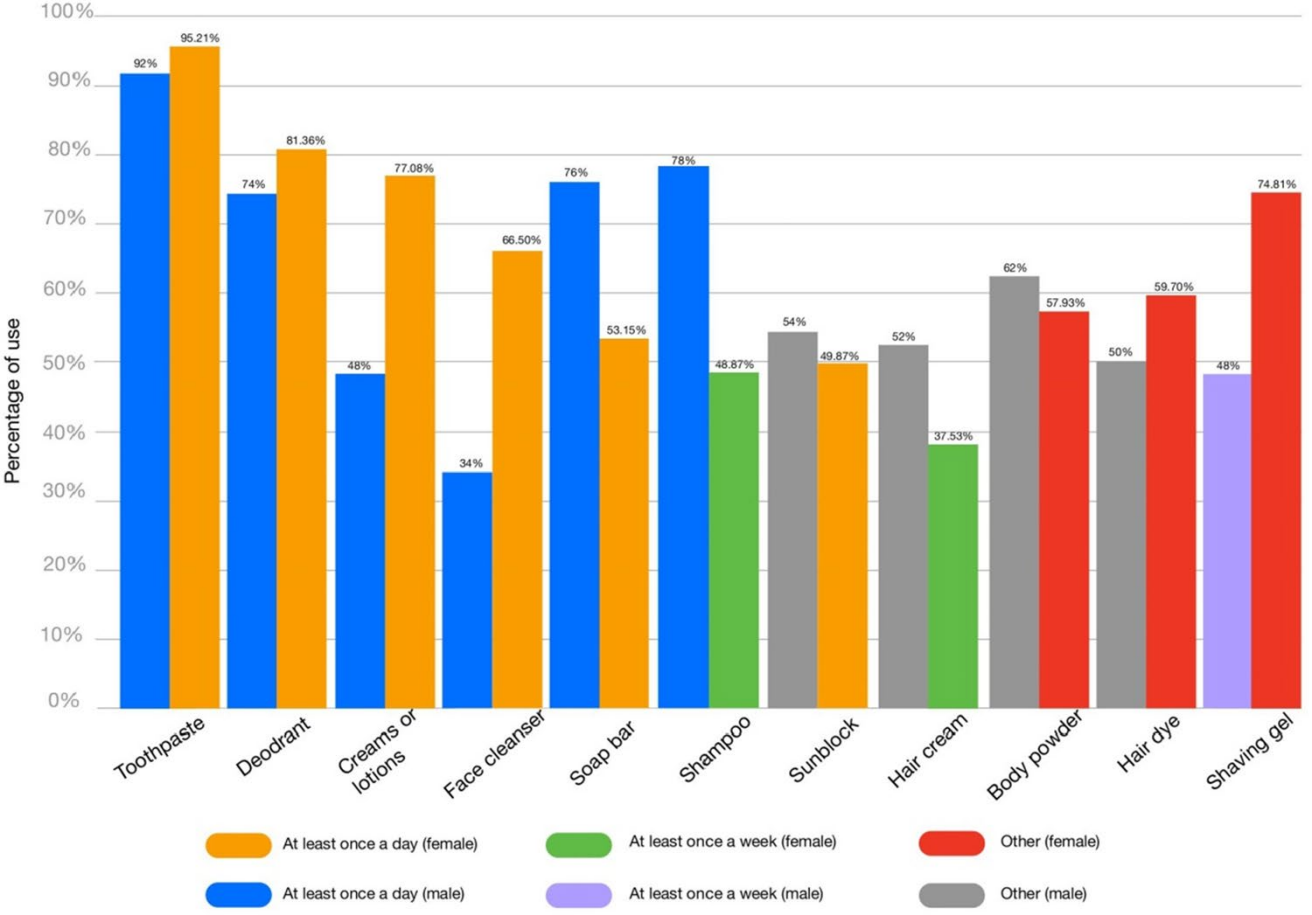
The most frequently used products according to the 447 participants.

Based on 447 responses from both males and females with different ages and education levels (Fig. 1), 21 products were chosen, including sunblock, BB cream, lip balm, hand cream, hair cream, shampoo, cleanser, baby oil, baby powder, soap bar, hair dye, makeup powder, makeup mousse cream, mascara, deodorant, hair serum, shaving gel, lipstick, toothpaste and lotion (Fig. 2). The products were coded and are presented in Table 1.

### Microbial load count

**Figure 3:**
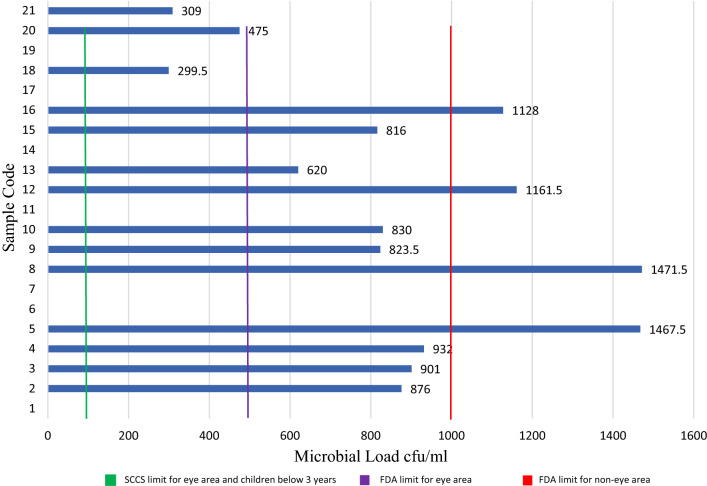
Microbial count results and permitted limits according to the FDA^14^ and SCCS^16^.

The mean microbial load count and microorganisms found in the topical cosmetic preparations are shown in Table 3 and Fig. 3. No microbial load was found in sunblock, shampoo, cleanser, hair dye, mascara, shaving cream or one of the kinds of toothpaste. Other samples yielded contaminated results, where the microbial load ranged from 1471.5 to 299.5 cfu/ml. Baby oil showed the highest contamination, 1471.5 cfu/ml, whereas lipstick showed the lowest result, 299.5 cfu/ml. *Staphylococcus aureus* (*S. aureus*) and *Bacillus* species were detected in the samples. *S. aureus* was found in all the samples, except for the BB cream and hand cream, as *Bacillus* species were predominant, with microbial loads of 876 cfu/ml and 932 cfu/ml, respectively (Table 3). Lip balm, makeup mousse cream, bar soap and deodorant yielded *S. aureus* alone, whereas the rest of the samples yielded both *S. aureus* and *Bacillus* species.

**Table 3:** Microbial counts and identification of the isolated microorganism(s).

Sample code	Mean counts (cfu/ml)	Isolated microorganism(s)
1	< 10	*–*
2	876	*Bacillus*
3	901	*S. aureus,*
4	932	*Bacillus*
5	1467.5	*S. aureus, Bacillus*
6	< 10	*–*
7	< 10	*–*
8	1471.5	*S. aureus, Bacillus*
9	823.5	*S. aureus, Bacillus*
10	830	*S. aureus*
11	< 10	*–*
12	1161.5	*S. aureus, Bacillus*
13	620	*S. aureus*
14	< 10	–
15	816	*S. aureus*
16	1128	*S. aureus, Bacillus*
17	< 10	–
18	299.5	*S. aureus, Bacillus*
19	< 10	*–*
20	475	*S. aureus, Bacillus*
21	309	*S. aureus, Bacillus*

### Heavy metals

The metal concentrations are presented in Table 4.

**Table 4:**
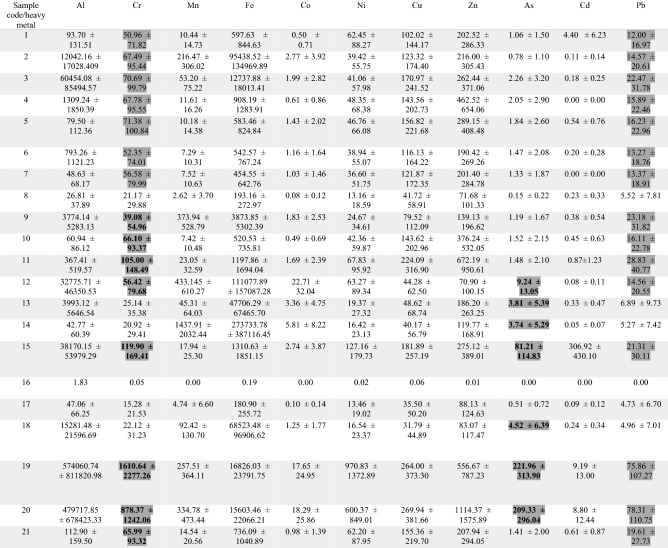
Heavy metal concentrations in ppm (mean ± SD) detected in the tested cosmetic products.

*Bolded and labeled in gray are the products that exceeded the US FDA limits.

### Organoleptic properties

The samples did not show lumps, grittiness, or discoloration, nor did they have a characteristic odor. The sample pH values ranged from 6.90 to 8.10.

## Discussion

**Figure 4:**
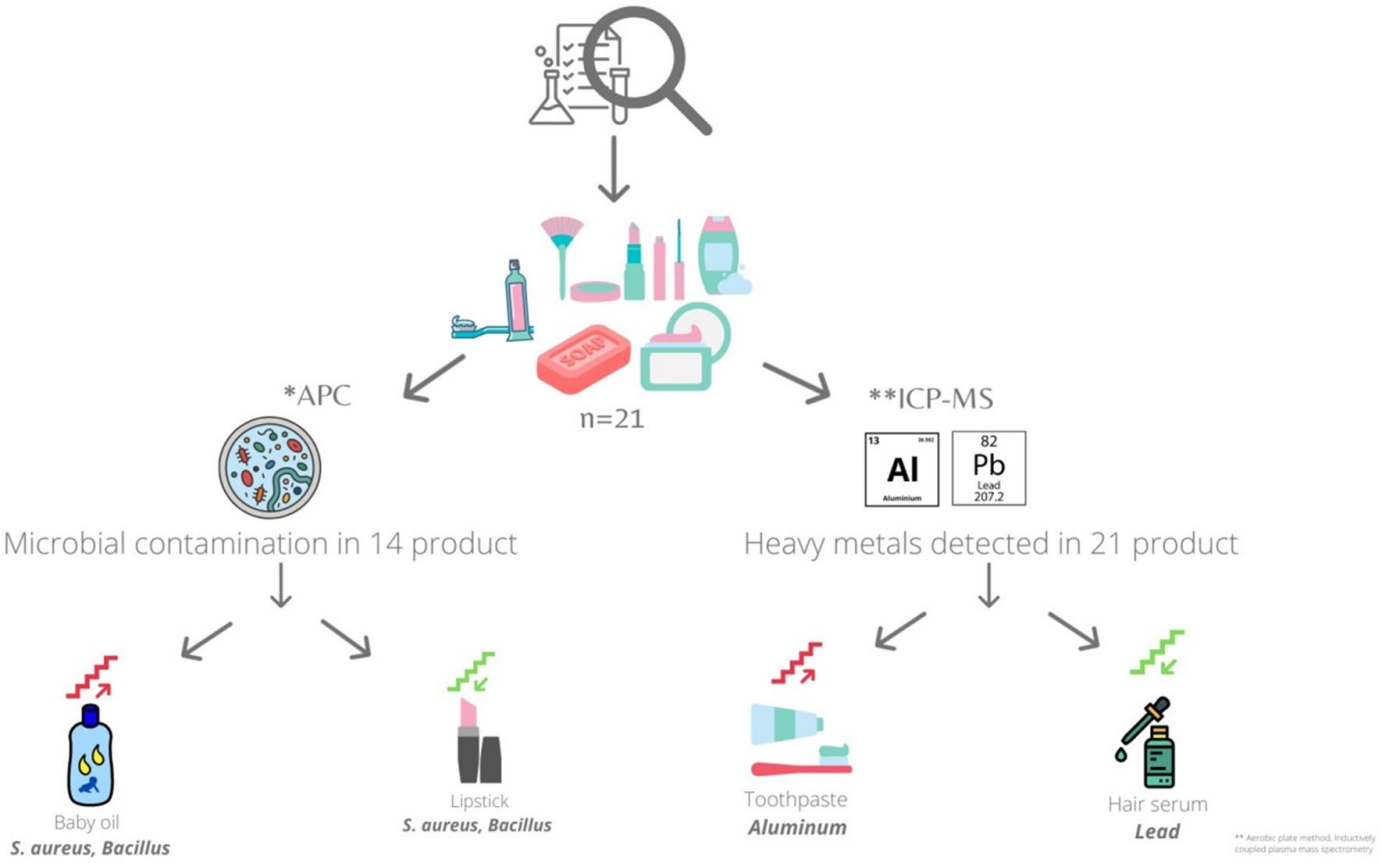
Graphical summary of this study.

Topical cosmetics are among the most consumed perorations worldwide. In this study, we explored the prevalence of cosmetics usage and examined some of the most common topical cosmetic products in terms of organoleptic properties, microbial load and heavy metal impurities (Fig. 4). This study showed that the participants used at least 5 products daily (Fig. 2). These preparations are intended to be in contact with several parts of the skin; thus, they can serve as vehicles for transmitting many pathogenic organisms^2^. The skin has defensive mechanisms that can protect the body from external matter; therefore, complete sterility is not essential for cosmetics. However, there is a threshold limit of microorganisms that can be handled by the skin^9^. This is especially true when cosmetics are applied around the eyes, on injured skin, on children under the age of three, on elderly individuals, and on compromised individuals.

The European Scientific Committee on Consumer Safety (SCCS) is a nonprofit organization composed of scientists from diverse professions who assess the safety of cosmetic components^16^. According to the SCCS ‘Notes of Guidance for Testing of Cosmetic Ingredients and Their Safety Evaluation,’ the microbial count must not exceed 100 (cfu)/g or ml for the products intended for children under 3 years or the products intended to be used in the eye area or mucous membranes. However, the microbial count should not exceed 1000 cfu/g for other products.

Moreover, the US FDA microbiological limits in cosmetics specify that the total number of microorganisms per gram or milliliter generally should not exceed 500 cfu/g for eye-area products and those for children < 3 years old, whereas the acceptable limit for all other topical cosmetics is ≤ 1000 cfu/g.

Both the SCCS and FDA consider the presence of opportunistic pathogens (*Escherichia coli* (*E. coli*), *S. aureus*, *Pseudomonas aeruginosa* (*P. aeruginosa*) or *Candida albicans* (*C. albicans*)) to be of significant concern.

Our results (Fig. 3) showed that both of the tested baby products exceeded the SCCS permitted limit. This is crucial since babies have a weak immune system, allowing the pathogen to easily enter their system if the skin is damaged^17^. This syndrome starts with irritability, fever, malaise, and a highly sensitive rash that appears within 24–48 h. Moreover, our study found that the makeup powder, hair cream and hair oil serum also exceeded the limit, with microbial loads of 1161.5 cfu/ml, 1467.5 cfu/ml and 1128 cfu/ml, respectively (Table 2).

In this study, 14 samples of the tested products showed microbial contamination. *S. aureus* was the most prominent microorganism found in 12 of the samples. In alignment with the study results, published studies have detected *S. aureus* as the main isolated microorganism in a variety of cosmetics items, in addition to other microorganisms, such as Candida, Rhodotorula, Salmonella, Citrobacter, Klebsiella, *Pseudomonas aerug*inosa and Alcaligenes^9,18–22^. *S. aureus* is a normal skin microorganism, but it may also act as an opportunistic pathogen that can cause a variety of skin and soft tissue infections^23^. Eczema, acne, erythematous rash and other skin infections can result from contaminated product usage^24^. The presence of *S. aureus* in eye products can cause infection to the external and internal tissues of the eye, including the tear duct, cornea conjunctiva, and posterior chambers, triggering profound damage that may lead to blindness^25^.

The other detectable microorganism that was found in 10 tested product samples was *Bacillus* species. *Bacillus* species are transient skin microflora. The group *Bacillu*s *cereus* is a *Bacillus genus* subdivision that currently includes eight formally recognized closely genetically related species that can cause focal necrotizing cellulitis in the skin^19^. *Bacillus* cereus contamination can lead to serious eye infections. This is especially true for cosmetics that are used on the eyes or near the eye, as found in this study in a makeup powder. This might be attributed to how powders easily come into contact with air and contain ingredients from a natural sources, such as talc in the formulation, which might increase the contamination level^19^. A published study showed that a *Bacillus cereus* with a microbial load as low as 100 cfu/ml caused infection in a susceptible animal model^26^. Abscesses, bacteremia/septicemia, wound and burn infections, ear infections, ophthalmitis, osteomyelitis, peritonitis, and respiratory and urinary tract infections have all been linked to *Bacillus* species. The majority of these diseases arise as secondary or mixed infections in immunocompromised individuals, but a high proportion are primary infections in healthy people^27^. In this study, we investigated the products as soon as they were opened, yet contamination was still found in some of the tested products. Most cosmetics are multiuse items that must retain low levels of contamination during consumer usage, which means that their preservation mechanisms must be efficient against contaminants that come into touch with the product after it has been opened^25^.

Heavy metal contamination in cosmetics is another serious issue. The Federal Food, Drug, and Cosmetic Act issued by the US suggests limits for some heavy metal impurities in cosmetics, such as lead, arsenic and chromium^28^. The maximum recommended limit of Pb as an impurity in cosmetics is 10 ppm. Cosmetic lip items (such as lipsticks, lip glosses, and lip liners) and externally applied cosmetics (such as eye shadows, blushes, shampoos, and body lotions) are covered by this guidance. The US FDA also limits the presence of Pb in cosmetic additives to 20 ppm. Our results showed that 6 of the tested cosmetic products exceeded this limit, and the highest value of Pb, 78.31 ppm, was found in sample 20 (toothpaste). Similarly, a study that investigated the same product and brand found but used atomic absorption spectrophotometry found that Pb exceeded the recommended limit with a value of 23.57 ppm^29^. In our study, we found that toothpastes were among the top most contaminated products that are used daily (Fig. 2). The accumulation of Pb by using lead-containing cosmetics regularly can result in serious side effects^30^. These side effects can vary from abdominal pain, headache and loss of appetite to more complicated manifestations, such as brain damage, renal dysfunction and paralysis^31,32^. Pregnant women can experience miscarriage or premature birth and give birth to babies with low birth weight^33^.

The US FDA has also set the limit for Cr in cosmetics additives to 50 ppm, and most of our tested cosmetic products exceeded this limit. In agreement with this finding, a study that tested nine of the most expensive brands of mascara and eye shadow from the Saudi market found that the level of Cr was higher than the acceptable limit in an eyeshadow product with a value of 7000 ppm^34^. Digestive, skin and ocular problems are linked to high exposure to Cr^35^.

Arsenic is limited to 3 ppm in cosmetics additives by the US FDA, and our tested cosmetic products exceeded this limit in samples 12 (makeup powder), 13 (makeup mousse cream), 14 (mascara), 15 (deodorant), 18 (lipstick), 19 (toothpaste), and 20 (toothpaste). The highest concentration of As was found in sample 19 (toothpaste) (221.96 ppm). Long-term exposure to arsenic might result in skin conditions such as lesions and skin, bladder and lung cancers^36^.

Since there are no limits set for other metals in cosmetics, such as Al, Mn, Fe, Co, Ni, Cu, and Zn, the US Pharmacopeia chapter <232> has established acceptability metal impurity criteria for drug products. Even though these guidelines are meant for drugs in different dosage forms, they may be relevant to avoid undesirable consequences. For example, Cd as an elemental impurity should be limited to 0.5 ppm, where in our tested cosmetic products, samples 1 (sunblock cream), 15 (deodorant), 19 (toothpaste), and 20 (toothpaste) greatly exceeded this limit (Table 3). The continued use of cadmium-contaminated products may cause heart diseases, hypertension, kidney and liver damage, and a weak immune system^37^.

The USP limits Co as an elemental impurity to 5 ppm, wherein tested cosmetic product samples 12 (makeup powder), 14 (mascara), 19 (toothpaste), and 20 (toothpaste) exceed this limit, and the highest value (22.71 ppm) was detected in sample 12 (makeup powder). Similarly, other cosmetic products exceeded the 5 ppm limit, such as an eyeliner (11.80 ppm)^38^. Long-term exposure to cosmetic products that are contaminated with Co may cause skin irritation^13^ Ni should not exceed 20 ppm according to the USP. Interestingly, most of the tested cosmetic products in this study exceeded this limit, and the highest value was detected in sample 19 (toothpaste). The major concern with nickel is allergic contact dermatitis, which can be a serious issue for patients with chronic eczema^39,40^. The only metal level that did not exceed the USP limit in any of the tested samples was Cu. This elemental impurity should be limited to 300 ppm, and all tested cosmetic products were below this value.

Unfortunately, there are no stated FDA or USP limits for other metals, such as aluminum, iron, zinc, and manganese. Short exposure to lower levels of manganese may lead to erythema and eczema, while chronic exposure has been linked to neurological disorders, such as loss of coordination and balance, forgetfulness, anxiety, and insomnia^40–43^. In this study, the highest concentrations of Mn and Fe were detected in sample 14 (mascara). Similarly, Aldayel et al.^34^ investigated nine of the most expensive brands of mascara and eyeshadow from the Saudi market and found that the concentration of Fe was high in the mascara products. Moreover, frequent use of cosmetics that contain aluminum has been linked to neurological disorders such as Alzheimer's disease, but the exact mechanism is unknown^40,44^. According to studies, high exposure to Zn, and Cu may lead to the interaction between them that can lead to abnormal Zn metabolism, so the balance between Zn and other nutrients could be destroyed^45^.

The highest concentrations of Al and Zn in this study were detected in sample 19 (toothpaste) and sample 20 (toothpaste), respectively. In alignment with this result, Al and Zn concentrations were high in different brands of toothpaste^31,46^.

Based on our tested cosmetic products, the highest levels of Pb, Cr, As, Ni, Zn, Al impurities were found in toothpaste. Interestingly, our results showed that toothpaste was the most frequently used cosmetic product in both sexes (Fig. 2). Therefore, both the quality and safety of cosmetics preparations must be more rigorously regulated.

Although there are some legal criteria to consider during cosmetics manufacture, this study showed that these regulatory procedures are not completely effective. Tests are required by authoritative agencies to ensure the safety and quality of cosmetic products or ingredients before they are put on the market. Moreover, the raw materials should be selected and handled properly by manufacturers.

## Conclusion

In this study, we investigated some cosmetic products to show the importance of quality and safety assurance. The study showed that many cosmetic products were used daily, and microbial and heavy metal contamination was found in many of the tested cosmetic products. The continuous use of such contaminated cosmetics can pose risks to human health. The manufacturers of cosmetic preparations must formulate and implement strict protocols for quality assurance, and these must be assessed and evaluated by regulators.

## Data Availability

All data generated or analysed during this study are included in this published article.

## Abbreviations

FDA: Food and Drug Administration
USFDA: United States Food and Drug Administration
SFDA: Saudi Food and Drug Authority
USP: United States Pharmacopeia
Ph.Eur.: European Pharmacopoeia
SCCS: Scientific Committee on Consumer Safety
SASO: Saudi standards, Metrology, and Quality Organization
API: Active Pharmaceutical Ingredient
GMP: Good Manufacturing Practice
TVC: Total viable count
CFU: Colony forming unit
*S. aureus*: *Staphylococcus aureus*
*P. aeruginosa*: *Pseudomonas aeruginosa*
*E. coli*: *Escherichia coli*
MRSA: Methicillin-resistant *S. aureus*
ICP-MS: Inductively coupled plasma mass spectrometer
Al: Aluminum
Fe: Iron
Zn: Zinc
Cr: Chromium
Mn: Manganese
Hg: Mercury
Cu: Copper
Ni: Nickel
As: Arsenic
Pb: Lead
Cd: Cadmium
Co: Cobalt
Ba: Beryllium
ppm: Parts per million
ppb: Parts per billion
SD: Standard deviation
